# Encouraging

**Published:** 1869-01

**Authors:** 


### EDITORIAL DEPARTMENT.

*Encouraging.—The St. Louis Medical Reporter* in noticing the *Balto. Medical Bulletin* thus speaks of the *American Journal of Dental Science:* "Surely Baltimore can support one medical journal. They have one of the best and most elegant dental journals published in the United States, and we would have thought that the medical profession could have sustained as good a medical work."

To know that our efforts are appreciated is certainly very encouraging, and a stimulus to continue and improve in well-doing.

